# Comparative genomics for mycobacterial peptidoglycan remodelling enzymes reveals extensive genetic multiplicity

**DOI:** 10.1186/1471-2180-14-75

**Published:** 2014-03-24

**Authors:** Edith Erika Machowski, Sibusiso Senzani, Christopher Ealand, Bavesh Davandra Kana

**Affiliations:** 1 DST/NRF Centre of Excellence for Biomedical TB Research, Faculty of Health Sciences, University of the Witwatersrand, National Health Laboratory Service, P.O. Box 1038, Johannesburg 2000, South Africa

**Keywords:** Peptidoglycan, Transglycosylases, Amidases, D,D-carboxypeptidases, Transpeptidases, Endopeptidases

## Abstract

**Background:**

Mycobacteria comprise diverse species including non-pathogenic, environmental organisms, animal disease agents and human pathogens, notably *Mycobacterium tuberculosis*. Considering that the mycobacterial cell wall constitutes a significant barrier to drug penetration, the aim of this study was to conduct a comparative genomics analysis of the repertoire of enzymes involved in peptidoglycan (PG) remodelling to determine the potential of exploiting this area of bacterial metabolism for the discovery of new drug targets.

**Results:**

We conducted an *in silico* analysis of 19 mycobacterial species/clinical strains for the presence of genes encoding resuscitation promoting factors (Rpfs), penicillin binding proteins, endopeptidases, L,D-transpeptidases and *N*-acetylmuramoyl-L-alanine amidases. Our analysis reveals extensive genetic multiplicity, allowing for classification of mycobacterial species into three main categories, primarily based on their *rpf* gene complement. These include the *M. tuberculosis* Complex (MTBC), other pathogenic mycobacteria and environmental species. The complement of these genes within the MTBC and other mycobacterial pathogens is highly conserved. In contrast, environmental strains display significant genetic expansion in most of these gene families. *Mycobacterium leprae* retains more than one functional gene from each enzyme family, underscoring the importance of genetic multiplicity for PG remodelling. Notably, the highest degree of conservation is observed for *N*-acetylmuramoyl-L-alanine amidases suggesting that these enzymes are essential for growth and survival.

**Conclusion:**

PG remodelling enzymes in a range of mycobacterial species are associated with extensive genetic multiplicity, suggesting functional diversification within these families of enzymes to allow organisms to adapt.

## Background

Bacteria inhabit every environment on earth with a resilience that is central to their survival and consequently, they continue to serve as a major source of human disease. A critical factor, which has been central to the success of these organisms, is the diversity entrenched within their cell walls, which serves as a major barrier to drug treatment. The mycobacterial cell wall is an incredibly complex structure, with multiple layers that collectively constitute a waxy, durable coat around the cell, which serves as the major permeability barrier to drug action [1-4]. Considering this, the cell wall and related components are attractive for the mining of new drug targets, and remain relatively unexploited for drug discovery in the case of certain bacterial pathogens [2,5,6]. Peptidoglycan (PG or the murein sacculus) is a rigid layer that constricts the cell membrane and the cell within, providing mechanical stability to counteract imbalances of cytoplasmic turgour pressure, and plays an important role in determining cell size and shape [7-10]. Mycobacteria possess a highly complex additional lipid rich outer membrane, with different constituents anchored either directly to the cell membrane or to the PG [6,11]. Arabinoglactan (AG), a structure unique to actinomycetes, is bound externally to an *N*-acetyl muramic acid (NAM) moiety of the PG [3,12]. In mycobacteria, a certain proportion of the muramic acid is *N*-glycolylated [13] through the activity of NamH, a UDP-*N*-acetylmuramic acid hydroxylase [14]. This modification results in altered tumour necrosis factor α production [15,16] however, abrogation of NamH activity does not lead to decreased virulence in mice [16].

**Figure 1:**
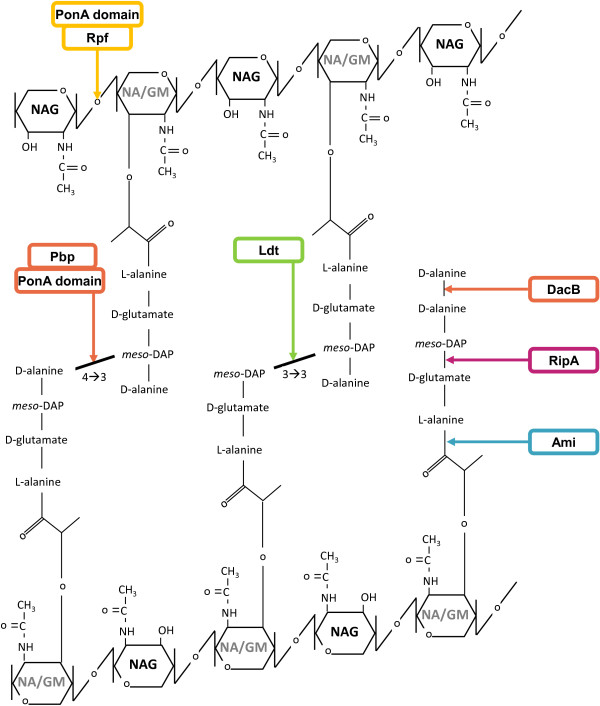
**PG units and chemical bonds associated with remodelling enzyme activities.** At the top and bottom of the figure are shown the NAG-NA/GM sugar backbone in anti-parallel orientation. The NAM residues are designated as NA/GM to correspond to the *N*-glycolylation of muramic acid in mycobacteria. Enzymatic activities are indicated by arrows: Rpfs [yellow], PBPs [orange], endopeptidases [pink], L,D-transpeptidases [green] and amidases [blue], which are related to the corresponding colours in Table 1. Amino acid residues in the stem peptide are shown in black text. Pentapeptide stems are attached to the Carbon at position 3 of the NAM ring. Transglycosylase activities of Rpfs and the Pon domain indicate their ß-1,4-glycosidic bond substrate. Synthetic enzyme activities are shown on the left, that is those that generate bonds cross-linking the pentapeptides on opposing stems, by Pon and Pbp proteins at positions 4,3 (L-Ala to *meso*-DAP) or Ldt proteins at positions 3,3 (*meso*-DAP to *meso*-DAP). The hydrolytic enzyme activities are shown to the right. These include the amidases, the RipA endopeptidases and the DD-CPase (DacB) acting on the pentapeptide stem (pre- or post-crosslinking).

This serves as an anchor for further lipid rich cell wall components, either by covalent attachment to the mycolic acid layer or through non-covalent interactions [trehalose dimycolate (TDM); phthiocerol dimycocerosate (PDIM); phenolic glycolipids (PGL)] [3,11,12]. PG consists of repeated alternating sugars *N*-acetyl glucosamine (NAG) and NA/GM (muramic acid with or without the glycolyl modification), which are linked to a pentapeptide side chain [7-9,17], Figure 1. The crosslinking of these subunits lead to a lattice-like structure around the cell.

The PG in bacterial cell walls is an incredibly dynamic structure that requires constant expansion and remodelling during growth to accommodate the insertion of new PG subunits, secretion apparatus, flagellae etc. [9,10]. During cell division, pre-septal PG synthesis and subsequent degradation of the septum is critical to daughter cell separation; consequently these processes are carefully regulated [7]. In this regard, there is a diversity of enzymes involved in cross-linking, degradation and remodelling of PG, which are illustrated in Figure 1. A ubiquitous feature in bacteria is the genetic multiplicity associated with these functions, which presumably contributes to the ability of different organisms to adapt under varying environmental conditions [7,9,10]. In the case of *Mycobacterium tuberculosis*, the causative agent of tuberculosis, there is a dire need for new drugs with novel modes of action. The increased prevalence of drug resistant strains has raised concerns regarding the sustainability of the current treatment regimen. To address this, several aspects of mycobacterial metabolism are being assessed for potential new drug targets [18]. The genetic redundancy associated with PG biosynthesis together with the reliance on robust bacterial growth to achieve significant drug target vulnerability, has hampered drug development initiatives that target the cell wall [19]. For other bacterial pathogens, PG has been successfully used as an antibiotic target in the past, as evidenced by the widespread use of β-lactam antibiotics among others, the biosynthesis and degradation of this macromolecule in mycobacteria is meritorious of further investigation.

In this study, we undertake a comprehensive analysis of the genomic repertoire of PG remodelling enzymes in various pathogenic and environmental mycobacteria to determine the level of genetic multiplicity/redundancy and degree of conservation. We focus on those enzymes involved in cross-linking and remodelling of the PG in the periplasmic compartment, including: resuscitation promoting factors (Rpfs), penicillin binding proteins (PBPs), transpeptidases, endopeptidases, and *N*-acetylmuramoyl-L-alanine amidases. Our data reveal extensive genetic multiplicity for the 19 strains analysed in this study, which allowed grouping of strains into three families based on their complement of PG remodelling enzymes, including the MTBC, other pathogenic mycobacteria and non-pathogenic environmental organisms.

## Results and Discussion

The comparative genomics analysis for PG remodelling enzymes in mycobacterial species obtained from this study is summarised in Table 1. We analysed 19 distinct species/strains: Six of these belong to the MTBC, six are classified as other pathogenic bacteria [three of which belong to the *Mycobacterium avium* complex (MAC)] and six environmental species including *Mycobacterium smegmatis. Mycobacterium leprae* is listed separately due to its substantially reduced genome which emerges as an outlier in the analysis.

**Table 1:** Genetic complement for PG remodelling enzymes in 19 mycobacterial species

	**MTB complex**						**Other mycobacterial pathogens**						**Environmental mycobacterial species**						
	***M. tuberculosis H37Rv***	***M. tuberculosis H37Ra***	***M. tuberculosis CDC1551***	***M. africanum GM041182***	***M. bovis AF2122/97***	***M. bovis *** **BCG Pasteur 1173P2**	***M. avium 104***	***M. avium subsp. paraTB K-10***	***M. intracellulare ATCC 13950***	***M. ulcerans AGY99***	***M. marinum M***	***M. abscessus ATCC19977***	***M. smegmatis *mc**^**2**^**155**	***M. vanbaalenii PYR–1***	***M. *sp. MCS**	***M. *sp. KMS**	***M. *sp. JLS**	***M. gilvum PYR–GCK***	***M. leprae TN***
Resuscitation promoting factors																			
*rpfA*	Rv0867c	MRA_0874	MT0890	MAF_08760	Mb0891c	BCG_0919c	MAV_0996	MAP0805c	OCU_08710	MUL_0283	MMAR_4665	MAB_0869c	MSMEG_5700	Mvan_5049	Mmcs_4479	Mkms_4566	Mjls_4862	Mflv_1703	ML2151
*rpfB*	***Rv1009***	MRA_1018	MT1038	MAF_10190	Mb1036	BCG_1066	MAV_1147	MAP0974	OCU_10320	MUL_4651	MMAR_4479	MAB_1130	MSMEG_5439	Mvan_4801	Mmcs_4264 Mmcs_1712	Mkms_4350 Mkms_1758	Mjls_ 4643 Mjls_1689	Mflv_1932	ML0240
*rpfC*	Rv1884c	MRA_1895	MT1932	MAF_19060	Mb1916c	BCG_1921c	MAV_2818	MAP1607c	OCU_26380	MUL_2975	MMAR_2772	MAB_4080	–	–	–	–	–	–	ML2030
*rpfD*	Rv2389c	MRA_2413	MT2458	MAF_24030	Mb2410c	BCG_2403c	–	–	–	–	–	–	–	–	–	–	–	–	–
*rpfE*	Rv2450c	MRA_2476	MT2526	MAF_24670	Mb2477c	BCG_2470c	MAV_1722	MAP2273c	OCU_18020	MUL_3723	MMAR_3776	MAB_1597	MSMEG_4643/MSMEG_4640	Mvan_3962/Mvan_3961	Mmcs_3564/Mmcs_3563	Mkms_3637/Mkms_3636	Mjls_3569/ Mjls_3568	Mflv_2619/Mflv_2620	–
Penicillin binding proteins																			
*ponA1*	*Rv0050*	MRA_0053	MT0056	MAF_00500	Mb0051	BCG_0081	MAV_0071	MAP0064	OCU_00670	MUL_0068	MMAR_0069	MAB_4901c	MSMEG_6900	Mvan_6036	Mmcs_5372	Mkms_5461	Mjls_5748	Mflv_0871	ML2688c
*ponA2*	Rv3682	MRA_3717	MT3784	MAF_36900	Mb3707	BCG_3741	MAV_0446	MAP0392c	OCU_03970	MUL_4257	MMAR_5171	MAB_0408c	MSMEG_6201 MSMEG_4384	Mvan_5442 Mvan_1068	Mmcs_4825 Mmcs_1483	Mkms_4911 Mkms_1505	Mjls_5212 Mjls_1480	Mflv_1365 Mflv_5209	ML2308
*pbpA*	*Rv0016c*	MRA_0018	MT0019	MAF_00160	Mb0016c	BCG_0046c	MAV_0020	MAP0019c	OCU_00180	MUL_0020	MMAR_0018	MAB_0035c	MSMEG_0031	Mvan_0025	Mmcs_0017	Mkms_0025	Mjls_0017	Mflv_0810	ML0018
*pbpB*	**Rv2163c**	MRA_2178	MT2221	MAF_21760	Mb2187c	BCG_2180c	MAV_2330	MAP1903c	OCU_22960	MUL_3508	MMAR_3200	MAB_2000	MSMEG_4233	Mvan_3529	Mmcs_3262	Mkms_3324	Mjls_3273	Mflv_2982	ML0908
*PBP-lipo*	Rv2864c	MRA_2889	MT2933	MAF_28690	Mb2889c	BCG_2886c	MAV_3723	MAP2936c	OCU_35570	MUL_2089	MMAR_1840	MAB_3167c	MSMEG_2584 MSMEG_6319	Mvan_2266 Mvan_4630	Mmcs_2047 Mmcs_4955	Mkms_2093 Mkms_5043	Mjls_2030 Mjls_5336	Mflv_4076 Mflv_2080	ML1577c
*dacB1*	Rv3330	MRA_3372	MT3433	MAF_33460	Mb3363	BCG_3400	MAV_4305	MAP3448	OCU_41630	MUL_1445	MMAR_1192	MAB_3681	MSMEG_1661	Mvan_1562	Mmcs_1216	Mkms_1233	Mjls_1243	Mflv_4869	ML0691
*dacB2*	Rv2911	MRA_2936	MT2979	MAF_29150	Mb2935	BCG_2932	MAV_3766	MAP2979	OCU_36070	MUL_2045	MMAR_1797	MAB_3234	MSMEG_2433/MSMEG_2432	Mvan_2184/Mvan_2183	Mmcs_1962/Mmcs_1961	Mkms_2008/Mkms_2007	Mjls_1942/Mjls_1941	Mflv_4179/Mflv_4180	–
Rv3627c	***Rv3627c***	MRA_3663	MT3729	MAF_36340	Mb3651c	BCG_3685c	MAV_0529	MAP0436	OCU_04440	MUL_4203	MMAR_5127	MAB_0519	MSMEG_6113	Mvan_5380	Mmcs_4778	Mkms_4864	Mjls_5164	Mflv_1409	ML0211
MSMEG_1900	–	–	–	–	–	–	–	–	–	–	–	MAB_2019	MSMEG_1900	Mvan_4520	Mmcs_0342	Mkms_0352	Mjls_0331	Mflv_2177	–
Endo-Peptidases																			
Rv0024	Rv0024	MRA_0027	MT0027	MAF_00240	Mb0024	BCG_0054	MAV_0042	MAP0036	OCU_00360	MUL_0042	MMAR_0043	–	–	–	–	–	–	–	–
*ripA*	**Rv1477**	MRA_1487	MT1524	MAF_15000	Mb1513	BCG_1539	MAV_3301	MAP1203	OCU_31420	MUL_1486	MMAR_2284	MAB_2728c	MSMEG_3145	Mvan_3656 Mvan_2747	Mmcs_1440 Mmcs_2451	Mkms_5716 Mkms_1458 Mkms_2496	Mjls_ 2488 Mjls_4564 Mjls_4520	Mflv_5292 Mflv_0895 Mflv_2839 Mflv_3663	ML1812
*ripB*	Rv1478	MRA_1488	MT1525	MAF_15010	Mb1514	BCG_1540	MAV_3300	MAP1204	OCU_31410	MUL_1487	MMAR_2285	MAB_2727c	MSMEG_3146	Mvan_2748 Mvan_3652	Mmcs_2452 Mmcs_1447	Mkms_2497 Mkms_1465 Mkms_5687 Mkms_5720	Mjls_ 2489 Mjls_ 4472 Mjls_ 4557 Mjls_ 4529	Mflv_5324 Mflv_5288 Mflv_0902 Mflv_2843	ML1811
*ripD*	Rv1566c	MRA_1578	MT1617	MAF_15930	Mb1593c	BCG_1619c	MAV_3208	MAP1272c	OCU_30430	MUL_1557	MMAR_2381	(MAB_2474)	(MSMEG_3477)	(Mvan_2970)	(Mmcs_2672)	(Mkms_2717)	(Mjls_2702)	(Mflv_3253)	ML1214
Rv2190c	*Rv2190c*	MRA_2205	MT2245	MAF_22010	Mb2213c	BCG_2206c	MAV_2304	MAP1928c	OCU_22720	MUL_3545	MMAR_3234	MAB_1974	MSMEG_4256	Mvan_3552 Mvan_3713	Mmcs_3287 Mmcs_1435	Mkms_3349 Mkms_5661 Mkms_1453	Mjls_3298 Mjls_4528 Mjls_4570	Mflv_2959 Mflv_5385 Mflv_5350 Mflv_2808 Mflv_0888	ML0885
L,D-transpeptidases																			
*ldt*_*Mt1*_	Rv0116c	MRA_0123	MT0125	MAF_01170	Mb0120c	BCG_0150c	MAV_5194	MAP3520c	OCU_50160	MUL_4806	MMAR_0316	MAB_3165c	MSMEG_3528	Mvan_3019	Mmcs_2729	Mkms_2773	Mjls_2759	Mflv_3298	ML2664
*ldt*_*Mt2*_	**Rv2518c**	MRA_2545	MT2594	MAF_25330	Mb2547c	BCG_2539c	MAV_1661	MAP2322c	OCU_17500	MUL_3804	MMAR_3872	MAB_1530	MSMEG_4745	Mvan_4102 Mvan_3651 Mvan_5854	Mmcs_1448 Mmcs_3641	Mkms_5721 Mkms_3714 Mkms_1466	Mjls_3646 Mjls_4532 Mjls_4556	Mflv_2542 Mflv_5287 Mflv_0904	ML0426
*ldt*_*Mt3*_	Rv1433	MRA_1442	MT1477	MAF_14550	Mb1468	BCG_1494	MAV_4834	MAP3812c	OCU_47330	–	MMAR_3552	MAB_4775	MSMEG_0674	–	–	–	Mjls_4515	Mflv_1397	ML0569
*ldt*_*Mt4*_	Rv0192	MRA_0200	MT0202	MAF_01930	Mb0198	BCG_0229	MAV_4986	MAP3634	OCU_48990	MUL_1085	MMAR_0435	MAB_4537c	MSMEG_0233	Mvan_3694 Mvan_0177	Mmcs_0151	Mkms_5680 Mkms_0160	Mjls_4535 Mjls_0141	Mflv_5330 Mflv_2824 Mflv_5369 Mflv_0479	–
*ldt*_*Mt5*_	*Rv0483*	MRA_0490	MT0501	MAF_04870	Mb0493	BCG_0524	MAV_4666	MAP3976	OCU_45320	MUL_4553	MMAR_0809	MAB_4061c	MSMEG_0929	Mvan_0824	Mmcs_0654	Mkms_0667	Mjls_0647	Mflv_0089	ML2446
Amidases																			
*ami1*	Rv3717	MRA_3754	MT3820	MAF_37260	Mb3744	BCG_3777	MAV_0385	MAP0318	OCU_03450	MUL_4308	MMAR_5233	MAB_0318c	MSMEG_6281	Mvan_5529	Mmcs_4905	Mkms_4994	Mjls_5273	Mflv_1286	ML2331
*ami2*	***Rv3915***	MRA_3954	MT4034	MAF_39300	Mb3946	BCG_0021	MAV_5303	MAP4341	OCU_51370	MUL_5068	MMAR_5479	MAB_4942	MSMEG_6935	Mvan_6069	Mmcs_5404	Mkms_5493	Mjls_5780	Mflv_0837	ML2704
*ami3*	Rv3811	MRA_3851	MT3918	MAF_38260	Mb3841	BCG_3873	MAV_0206	MAP0209c	OCU_02160	MUL_4995	MMAR_5375	MAB_0168c	MSMEG_6406	Mvan_5652	Mmcs_5022	Mkms_5110	Mjls_5403	Mflv_1157	–
*ami4*	Rv3594	MRA_3633	MT3700	MAF_36070	Mb3625	BCG_3659	–	–	–	–	–	MAB_4807	MSMEG_5315	Mvan_3376	Mmcs_4180	Mkms_4246	Mjls_4402	Mflv_3152	–

The names of the various organisms analysed are shown in the columns and gene complement is given in the corresponding rows. Mycobacteria are grouped as *M. tuberculosis* Cluster (MTBC), other pathogens, environmental species and *M. leprae*. Genes are sorted by functional groups in rows. The listing of a gene is based on its presence by protein BLAST analysis, either at curated sites or directly at NCBI. For all genes the protein sequence, in FASTA format, was obtained and utilised for phylogeny. Annotations for *M. africanum* (MAF_) and *M. intracellulare* (OCU_) were obtained directly from NCBI. BLAST analysis was performed against individual strains at NCBI using *M. tuberculosis* H37Rv homologues as the query sequence. The cut off was taken at a coverage of >90% and an identity of >40%. MSMEG_1900 was identified at SmegmaList. In the case of *ripD*, parentheses indicate the 63C-terminal amino acid truncation. Further in-depth information, and confirmation of gene annotation, was obtained by assessment of phylogeny based on protein sequences, Additional file 1 Figure S1-S7. Font differences in the *M. tuberculosis* H37Rv column indicate genes that have been annotated as essential by two different TraSH analyses – indicated in bold (Sassetti et al. [20]) and/or italicised (Griffin et al. [21]) are those genes identified as essential or required for optimal growth.

### Resuscitation promoting factors (lytic transglycosylases)

**Figure 2:**
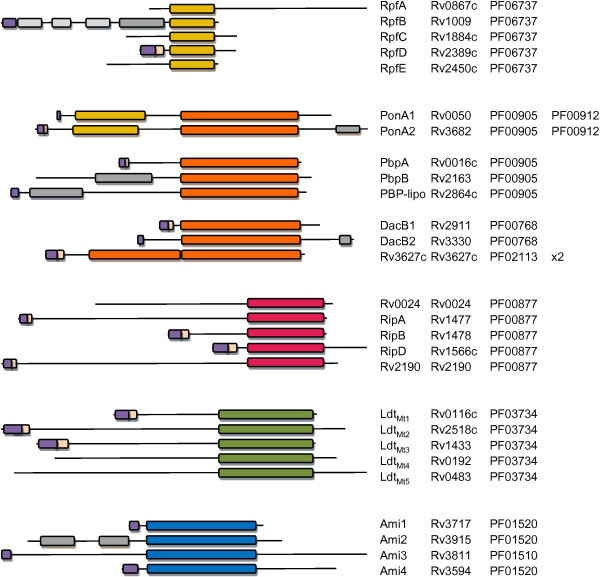
**Alignment and domains of *M. tuberculosis *H37Rv PG remodelling enzymes.** Domain architecture is based on output from InterScanPro. All enzymes depicted are the *M. tuberculosis* H37Rv homologues. Amino acid sequences are grouped according to their common domains, as indicated by their colors: Rpf domains [yellow], PBPs [orange], endopeptidases [pink], LD-transpeptidases [green] and amidases [blue]. PonA proteins are grouped with PBPs. PFAM domains are annotated as follows: PF06737 Transglycosylase-like domain, PF00905 PBP transpeptidases domain, PF00912 Transglycosylase domain, PF00768 D-alanyl-D-alanine Carboxypeptidase domain, PF02113 D-Ala-D-Ala carboxypeptidase 3 (S13) family domain, PF00877 NlpC/P60 family domain, PF03734 L,D-transpeptidase catalytic domain, PF01520 *N*-acetylmuramoyl-L-alanine amidase amidase_3 domain, PF01510 *N*-acetylmuramoyl-L-alanine amidase amidase_2 domain. N-terminal signal sequence or transmembrane domains are displayed as purple and pink, respectively. Additional domains annotated at PFAM are as follows (in grey): PonA2, PF03793, PASTA domain; PbpB, PF03717, PBP dimerization domain; PBP-lipo, PF05223, NTF2-like N-terminal transpeptidase; Ami2, PF01471, Peptidoglycan-binding like; RpfB, PF03990, Domain of unknown function DUF348; RpfB, PF07501, G5 domain. Rv3627c retains two tandem copies of the PF02113 D-Ala-D-Ala carboxypeptidase 3 (S13) family domain, one of which is contracted. Figure not to scale.

Of all the enzymes identified in this study, the Rpf family is the most extensively studied. This group of enzymes are of particular interest due to demonstrated importance for reactivation from dormancy and essentiality for growth in *Micrococcus luteus*[22,23]. Whilst *Mi. luteus* encodes a single, essential *rpf* gene, mycobacteria encode a multiplicity of *rpf* homologues and those present in *M. tuberculosis*, designated as *rpfA*-*rpfE*, encode closely related proteins all of which retain the Rpf domain [24-26], Figure 2. These have been the subject of intense study due to the potential role they may play in reactivation disease in individuals that harbour latent TB infection [25,27-31]. In this regard, the five *rpf* genes present in *M. tuberculosis* are collectively dispensable for growth but are differentially required for reactivation from an *in vitro* model of non-culturability [32,33]. Furthermore, the Rpfs are combinatorially required to establish TB infection and for reactivation from chronic infection in mice [32-35]. For additional information, the reader is referred to several extensive reviews on this topic [25,27,28,36-38].

Rpfs are classified as lytic transglycosylases (LTs) based on sequence conservation and three-dimensional protein structure [29,39-41]. LTs cleave the ß-1,4-glycosidic bonds between the NAG-NA/GM sugar subunits, Figure 1, and their activity is required for insertion of new PG units and expansion of the glycan backbone [9]. In mycobacteria RpfB contains a lysozyme-like, transglycosylase-like PFAM domain, and consequently this group of enzymes are predicted to cleave the glycan backbone of PG [39-41]. Direct evidence for this is lacking and moreover, the mechanism through which Rpf-mediated cleavage of PG results in growth stimulation remains unknown. The repertoire of *rpf* genes is highly conserved in the MTBC; in contrast, other pathogenic mycobacteria lack *rpfD*, including *M. leprae*, Table 1. Based on the distribution of *rpfC* and *rpfD*, we categorize the 19 strains analysed in this study into the MTBC (which retains all five *rpf* homologues present in *M. tuberculosis*), other pathogenic mycobacteria (which lack *rpfD*) and environmental strains (which lack both *rpfC* and *rpfD*). This classification is supported by phylogenetics analysis which confirms these clusters and duplication/loss of genes, Additional file 1: Figure S1. Recently, it has been shown that the Rpfs can serve as potent antigens [42] and Rpf-directed host immune responses allow for detection of TB in latently infected individuals [43]. It is noteworthy that strains lacking different combinations of *rpf* genes confer significant protective efficacy when used as vaccine strains in mice [44]. Hence, any variation in *rpf* gene complement between pathogenic mycobacteria may have significant consequences for broadly protective effects of future Rpf-based vaccines.

The environmental species retain three *rpf* genes [*rpfA*, *rpfB* (duplicated in *Mycobacterium* sp. JLS, *Mycobacterium* sp. KMS, *Mycobacterium* sp. MCS) and *rpfE*], Table 1 and Additional file 1: Figure S1. Although *rpfC* (Rv1884c in *M. tuberculosis*) homologues have been annotated as present in all mycobacteria [45], our analysis shows that the *M. tuberculosis rpfC* homologue is absent from environmental species. Artemis Comparison Tool (ACT) whole genome alignment reveals that the region encoding *rpfC* in *M. tuberculosis* is absent in *M. smegmatis* and all other environmental mycobacteria (data not shown). Thus, based on gene synteny, there is no direct *rpfC* homologue in these strains. However, there is a local duplication of *rpfE* in all the environmental strains (annotated as MSMEG_4643 in *M. smegmatis*), Table 1, Additional file 1: Figure S1. Consequently, we re-annotate MSMEG_4640 to *rpfE2*, as a homologue of MSMEG_4643, rather than a homologue of *Rv1884c*. As RpfE interacts with the Rpf Interacting Protein A (RipA) [46], there may be some functional consequence to the presence of multiple copies in *M. smegmatis* and other environmental bacteria.

The restriction of *rpfC* and *rpfD* homologues to pathogenic and MTBC strains, along with the duplication of *rpfB* in some environmental species, raises interesting questions regarding the nature of growth stimulation in these organisms. These differences suggest that the latter require fewer secreted Rpfs and are more reliant on the membrane bound RpfB homologue. This could be related to the fact that environmental organisms are required to grow in diverse niches of varying size and complexity making them more dependent on localised growth stimulatory activity through a membrane bound Rpf rather than paracrine signalling from diffusible Rpfs produced by neighbouring organisms. It is noteworthy that of all five homologues in *M. tuberculosis*, deletion of *rpfB* individually or in combination with *rpfA* results in colony forming defects and prolonged time to reactivation from chronic infection in mice [21,34,35].

The role of Rpfs in TB disease in humans remains enigmatic. It has been demonstrated that sputum from patients with active TB disease, before the initiation of treatment, is characterised by a population of dormant bacteria that require Rpfs for growth [47]. These data provide tantalizing preliminary evidence that Rpfs play an important role in determining bacterial population dynamics in TB infected patients and moreover are critical for disease transmission. Within the granulomatous environment, it may be preferable for the bacterial population as a whole to facilitate emergence of fitter clones which are able to exit from arrested growth. This could explain clonal emergence in clinical samples if few strains are able to expand sufficiently to cause tubercular lung disease.

### Penicillin binding proteins

Penicillin Binding proteins (PBPs) are a large family of evolutionarily related cell wall associated enzymes, that bind β-lactam antibiotics [48,49]. PBPs are classified according to their molecular weight as either high molecular mass (HMM) or low molecular mass (LMM) and are broken down into Class A, Class B and Class C [49]. In mycobacteria, Class A PBPs constitute bi-functional enzymes designated as *ponA1* (PBP1, Rv0050, [50]); and *ponA2* (PBP1A, Rv3682 [51]), Figure 2. They contain separate domains for transpeptidase and transglycosylase activities. Both these genes are present in all mycobacteria and, as previously reported for *M. smegmatis* and other environmental strains, there is a duplication of *ponA2* which was annotated as *ponA3*[51], Table 1 and Additional file 1: Figure S2.

Class B PBP proteins PbpA (*pbpA*; Rv0016c, [52]), PbpB (*pbpB*; Rv2163c, [53]) and PBP-lipo (Rv2864c, [49]) are predicted to contain only transpeptidase domains and possibly additional dimerisation domains, but lack transglycosylase activities, Figure 2. Both PbpA and PbpB (FtsI) are involved in progression to cell division in *M. smegmatis* where gene deletion or depletion manifests in altered cell morphology and antibiotic resistance profiles [52]. In this family of PBPs – as exemplified by *ponA2* - there is a distal duplication of PBP-lipo in the environmental strains, Table 1 and Additional file 1: Figure S3. No experimental data on this are currently available, but the lipophilic domain is speculated to allow for cell wall association.

D,D-carboxypeptidases (DD-CPases) are designated as Class C PBPs and are generally present in high abundance [54]. DD-CPases remove the D-Ala residue at position 5 of pentapeptides [8] and through this activity prevent cross linking of the stem peptide into 4 → 3 bridges, Figure 1. In mycobacteria, the *dacB2*-encoded DD-CPase is not affected by penicillin – though it does bind the antibiotic [55]. Inhibition of DacB through treatment with meropenem results in the accumulation of pentapeptides in *M. tuberculosis*[56]. In this context, DD-CPases have been implicated in regulating the amount of cross-linking that can occur within the PG sacculus [8]. Our analysis shows that *M. tuberculosis* H37Rv encodes three distinct DD-CPase homologues: *dacB1* (Rv3330), *dacB2* (Rv2911) and Rv3627c, Table 1, Figure 2 and Additional file 1: Figure S4. Rv3627c carries two PF02113 domains, one of which is contracted. In the environmental species there is a local duplication of the *dacB2* (Rv2911) homologue, leading to consecutive numbering of the resulting duplicated genes for example, MSMEG_2432 and MSMEG_2433 in *M. smegmatis*. In addition, a distant DD-CPase homologue (annotated as MSMEG_1900 in *M. smegmatis*) was identified in the environmental strains, as well as in *M. abscessus* but not in the other pathogenic mycobacteria and MTBC, Table 1. Two additional loci - Rv0907 and Rv1367c – were identified in *M. tuberculosis* by *in silico* analysis through their predicted ß-lactamase domains and are grouped among Class C PBPs [49]. Analysis of these proteins revealed that they retain a β-lactamase binding domain (of the AmpH family) but further classification into the functional classes studied herein proved difficult. Consequently, we have not analysed these genes further.

### Endopeptidases

Endopeptidases are enzymes that cleave within the stem peptides in PG. In this study, we focus on the Nlp/P60 class of endopeptidases, which cleave within the stem peptides between positions 2 and 3 as exemplified by RipA, Figure 1. RipA is an essential PG hydrolytic enzyme that synergistically interacts with RpfB and RpfE [46,57] to form a complex that is able to degrade PG. The RipA-RpfB hydrolytic complex is negatively regulated by PonA2 [58] suggesting a dynamic interplay between PG hydrolases, one that would be significantly nuanced with the presence of multiple RipA and Rpf homologues. In this regard, our analysis reveals four endopeptidases in *M. tuberculosis* that display strong homology to *ripA*, Table 1, Figure 2, Additional file 1: Figure S5. With the exception of *Mycobacterium abscessus* and *M. leprae*, pathogenic mycobacteria retain all five of these homologues. Environmental strains display enhanced expansion of endopeptidases, with the exception of the *ripD* homologue (Rv1566c). The functional consequence of this remains unknown but it is noteworthy that these strains have also expanded their *rpfE* and *rpfB* gene repertoire, suggesting that the multiplicity in this case allows for a greater number of RipA-RpfB/E protein complexes, as well as for protein complexes with different subunit composition. Dysregulated expression of RipA leads to dramatic alterations in cellular morphology and growth [59] suggesting that careful regulation of this protein, both at the expression level as well as by post-translational level is essential. Genetic expansion of RipA homologues along with two copies of RpfB and RpfE, both of which interact with RipA implies a functional consequence of this expansion. In addition, strong regulation of these multiple copies would be required to prevent any detrimental effects on cell growth.

RipB displays strong sequence homology RipA in *M. tuberculosis* (100% amino acid identity over 58% coverage) and similar domain organization [60], but lacks the N-terminal motif, Figure 2, that has been implicated in auto inhibition by blocking the active site in the three-dimensional crystal structure [61]. More recently, high resolution crystal structures of RipB and the C-terminal module of RipA (designated as RipAc) revealed striking differences in the structure of these proteins, specifically in the N-terminal fragments that cross the active site [60]. Both RipB and RipAc are able to bind high molecular weight PG and retain the ability to cleave PG with variable substrate specificity, which is not regulated by the presence of the N-terminal domain [60]. This suggests that the N-terminus does not regulate PG degrading activity and in this context, the physiological consequences of the reduced size of RipB and RipD, Figure 2, remain unknown. The high degree of conservation of RipB across all pathogenic mycobacteria including *M. leprae*, Table 1, Additional file 1: Figure S5 indicates that variable substrate specificity in PG hydrolases in essential for pathogenesis. The *Mycobacterium marinum* homologues of Rv1477 and Rv1478, *iipA* and *iipB* (MMAR_2284 and MMAR_2285 respectively), Table 1, Additional file 1: Figure S5, have been implicated in macrophage invasion, antibiotic susceptibility and cell division [62]. As with the other enzymes assessed in this study, environmental mycobacteria display greater genetic multiplicity for these homologues, Table 1.

Structural analysis of RipD reveals alterations in the catalytic domain, consistent with the inability of this protein to hydrolyse PG [63]. Nevertheless the core domain of RipD is able to bind mycobacterial PG and this binding is negatively regulated by the C-terminal region [63]. However, RipD homologues in the environmental mycobacteria lack the 63C-terminal amino acids, Table 1 (shown in parentheses), possibly allowing for stronger binding of this enzyme to PG.

Rv2190c encodes another NlpC/P60-type PG hydrolase in mycobacteria. Deletion of this gene in *M. tuberculosis* results in altered colony morphology, attenuated growth *in vitro*, defective PDIM production and reduced colonisation of mouse lungs in the murine model of TB infection [64]. Consistent with this, homologues of Rv2190c are found in all pathogenic mycobacteria, Table 1, with notable genetic expansion in some environmental species. In contrast, the Rv0024 is absent from environmental species, suggesting that it could be required for intracellular growth or some other component of the pathogenic process, Table 1, Additional file 1: Figure S5.

### L,D - Transpeptidases

L,D-transpeptidases (Ldt) are a group of carbapenem sensitive enzymes in *M. tuberculosis*[56] that contribute to the formation of a 3 → 3 link between the two adjacent mDAP (mDap → mDap bridges) residues in PG, distinct from the classic 4 → 3 link (D-Ala → mDAP), Figure 1. *M. abscessus*[65] and *M. tuberculosis*[66] exhibit increased ratios of the 3 → 3 cross-link in stationary axenic culture, indicating that mycobacteria are capable of modulating their PG at the level of transpeptidation in response to growth stage and the availability of nutrients. Both Ldt_Mt1_ and Ldt_Mt2_ (Rv0116c and Rv2518c respectively) were experimentally shown to affect *M. tuberculosis* H37Rv morphology, growth characteristics and antibiotic susceptibility *in vivo*[67]. The crystal structure of Ldt_Mt2_ places the extramembrane domain 80–100 Å from the membrane surface and indicates that this enzyme is able to remodel PG within this spatial region of the PG sacculus [68]. More recently, it has been demonstrated that the combinatorial loss of both Ldt_Mt1_ and Ldt_Mt2_ in *M. tuberculosis* resulted in morphological defects and altered virulence in the murine model of TB infection [69]. A notable variability of L,D-transpeptidase genes is found in mycobacteria, Table 1, Figure 2 and Additional file 1: Figure S6. Five homologues are present in all but one pathogenic strain, while multiple homologues are evident in most environmental strains. The exception is *ldt*_*Mt3*_ (Rv1433), which is absent from the pathogen *Mycobacterium ulcerans* and from the environmental species *Mycobacterium vanbaalenii*, *M* sp. MCS and *M.* sp. KMS, yet its presence in *M. leprae* suggests functional importance. As with RipA, *M. gilvum* shows the greatest expansion of the *ldt* genes. Biochemical characterisation of all five *M. tuberculosis* H37Rv homologues, Ldt_Mt1_ - Ldt_Mt5_, confirms PG cross-linking and/or ß-lactam acylating enzyme activities in all of these enzymes [70]. This activity can be abolished by treatment with imipenem and cephalosporins, indicating that this group of enzymes holds great promise for TB drug development [70,71]. Moreover, the functionality of all the Ldt homologues present in *M. tuberculosis* raises interesting questions with respect to the functional consequences of the expansion of this protein family in environmental strains, which may require greater flexibility in Ldt function.

### Amidases

While endopeptidases and transpeptidases are responsible for cleavage within or between peptide stems, amidases act to remove the entire peptide stem from the glycan strands, cleaving between the NA/GM moiety and the L-Ala in the first position of the stem peptide, Figure 1. The amidases have been implicated in PG degradation, antibiotic resistance/tolerance and cell separation in *Escherichia coli* and other organisms, and can be organised into 2 main families containing either an amidase_2 or amidase_3 – type domain [8,9,72]. The amidases of *E. coli* (which retains 5 amidases designated AmiA, AmiB, AmiC, AmiD and AmpD) have specific substrate requirements governed by the structural confirmation of the NAM carbohydrate moiety. Knockout of these amidases results in chaining phenotypes, abnormal cell morphologies and/or increased susceptibility to certain antibiotics [72-74]. Amidases have also been implicated in spore formation, germination and cell communication in *Bacillus subtilis*[75,76]. The role of amidases in mycobacterial growth, virulence and resuscitation from dormancy is unknown and any impact of these on mycobacterial morphology and antibiotic resistance remains to be demonstrated. Analysis of the amidase gene complement in mycobacteria reveals the presence of four homologues in *M. tuberculosis*, two containing the amidase_2 domain (*ami3*; *Rv3811* and *ami4*; *Rv3594*) and two the amidase_3 domain (*ami1*; *Rv3717* and *ami2*; *Rv3915*), Table 1, Figure 2 and Additional file 1: Figure S7. The crystal structure of Rv3717 from *M. tuberculosis* confirms that this enzyme is able to bind and cleave muramyl dipeptide [77]. The amidase family distinguishes itself from all other enzyme families by absence of a homologue (*ami4*) from non-MTBC pathogens and its presence in the MTBC and environmental strains. *M. leprae* retains only the *ami1* and *ami2* genes – both containing the amidase_3 domain. This suggests that amidase_2 domain amidase activity is dispensable specifically in this species, but required for peptidoglycan remodelling in the other pathogenic mycobacteria.

### Mycobacterium leprae

Very little is known about *in vitro* growth and division of *M. leprae*, as it can only be grown in animal models. From our analysis, it is apparent that *M. leprae* habours notable genetic redundancy for PG remodelling enzymes (Table 1) in contrast to its minimal gene set for other areas of metabolism [78]. Considering that PG subunits or precursors cannot be scavenged from the host, it is expected that pathogenic bacteria would retain complete pathways for biosynthesis and remodelling of PG. However, the presence in *M. leprae* of multiple homologues within each class of PG remodelling enzyme assessed in this study, suggests that some level of multiplicity is required to ensure substrate flexibility. Further work in this regard is difficult due to the limited tractability of *M. leprae* for *in vitro* manipulation.

## Conclusions

Mycobacteria represent a wide range of species with a great variety of phenotypes. Exposure to stresses which they encounter at various stages of their life cycles demands the ability to adapt. Consistent with this, many mycobacteria encode a multiplicity of genes for numerous important pathways such as respiration and cofactor biosynthesis [79,80], which allows for a more nuanced regulation of physiology. The analysis performed herein summarises the general distribution of PG remodelling genes in diverse strains and reveals an emerging trend towards gene multiplicity in environmental mycobacteria. There is great conservation within the MTBC and other pathogenic mycobacteria. Of all strains, *M. gilvum* displays the greatest degree of gene expansion, containing a total 44 PG remodelling genes, Table 1. This organism has not been studied extensively but may represent a potential model system for understanding how the genetic multiplicity for PG remodelling enzymes contributes to bacterial physiology. As expected *M. leprae* shows a reduction in the number of genes that encode the enzymes assessed in this study but still retains more than one representative of each functional class. This, together with the striking degree of conservation in some families of PG remodelling enzymes in pathogenic mycobacteria, suggests that PG biosynthesis, remodelling and possibly recycling are all potential vulnerable pathways for drug development. The extracellular nature of these enzymes provides an added advantage for drug screening since small molecules need not enter the cell for biological activity. Entry of compounds into mycobacterial cells remains the major confounding factor in current drug development initiatives. Moreover, the lack of human counterparts would ensure a high degree of specificity. In conclusion, the gene complements for PG remodelling revealed in this study most likely reflect the differential requirements of various mycobacteria for murein expansion/turnover during colonisation of and proliferation within host organisms or environmental niches.

## Methods

The 19 mycobacterial strain sequences used in this study were all complete and either published [24,78,81-90] or directly submitted to GenBank [91] (Additional file 2: Table S1). The following sites were utilized for analysis of the genomes (Additional file 2: Table S2): The comparative genomic profile for the enzymes of interest were initiated by homology searches of known *M. tuberculosis* H37Rv genes at TubercuList [92], GenoList [93] or TBDB [94]. Where necessary for further analysis direct BLAST analysis was performed at NCBI [95], utilising protein sequence for BLASTp or DNA sequence for BLASTn particularly for the analysis of *Mycobacterium* sp. JLS, *M. africanum* and *M. intracellulare* which are not or only partially annotated at TBDB. To confirm the absence of genes, protein sequence was used for tBLASTn analysis. Additional homologues that are absent from *M. tuberculosis* H37Rv were identified by advanced search at SmegmaList (Mycobrowser) [96]. Where information was required for sequence level analysis, the Sanger Artemis Comparison Tool (ACT) [97] was utilized on annotated sequences obtained from the Integrated Microbial Genomes (IMG) site at the DOE Joint Genome Institute [98]. Phylogeny was established from FASTA files from all genes in Table 1 at EMBL-EBI by ClustalO [99] alignment and ClustalW2 [100] analysis and visualized using FigTree V1.4 software (http://tree.bio.ed.ac.uk/software/figtree). Functional annotation of each of the *M. tuberculosis* proteins was identified at InterScanPro [101], for PFAM domains [102], signal sequences (SignalP) [103] and membrane anchoring domains (TMHMM) [104].

## Competing interests

The authors declare that they have no competing interests.

## Authors’ contributions

BDK conceived and designed the study. EEM conducted all the bioinformatics analyses and compiled the manuscript. SS and CE provided intellectual input on certain aspects of the study. All authors approve of the final content in the manuscript.

## Supplementary Material

Additional file 1: Figure S1 Phylogenetic relationship between Resuscitation Promoting Factors from various mycobacteria. **Figure S2**. Phylogenetic relationship between Class A penicillin binding proteins (PonA family) from various mycobacteria. **Figure S3**. Phylogenetic relationship between Class B penicillin binding proteins (Pbp family) from various mycobacteria. **Figure S4**. Phylogenetic relationship between Class C penicillin binding proteins (DD-carboxypeptidases) from various mycobacteria. **Figure S5**. Phylogenetic relationship between endopeptidases (Nlp/P60 – domain containing proteins) from various mycobacteria. **Figure S6**. Phylogenetic relationship between L,D-transpeptidases from various mycobacteria. **Figure S7**. Phylogenetic relationship between N-acetylmuramoyl-L-alanine from various mycobacteria. Click here for file

Additional file 2: Table S1 Mycobacterial strains included in this study. **Table S2**. Bioinformatics sites used for analysis. Click here for file
