# Supplementary material for: Comparative genomics for mycobacterial peptidoglycan remodelling enzymes reveals extensive genetic multiplicity
Source: BMC Microbiol. 2014 Mar 24;14:75. doi: 10.1186/1471-2180-14-75 (PMC3987819; doi:10.1186/1471-2180-14-75)
Supplement: Additional file 1: Figure S1 — Phylogenetic relationship between Resuscitation Promoting Factors from various mycobacteria. Figure S2. Phylogenetic relationship between Class A penicillin binding proteins (PonA family) from various mycobacteria. Figure S3. Phylogenetic relationship between Class B penicillin binding proteins (Pbp family) from various mycobacteria. Figure S4. Phylogenetic relationship between Class C penicillin binding proteins (DD-carboxypeptidases) from various mycobacteria. Figure S5. Phylogenetic relationship between endopeptidases (Nlp/P60 – domain containing proteins) from various mycobacteria. Figure S6. Phylogenetic relationship between L,D-transpeptidases from various mycobacteria. Figure S7. Phylogenetic relationship between N-acetylmuramoyl-L-alanine from various mycobacteria. [file 1471-2180-14-75-S1.pptx]

## Slide 1
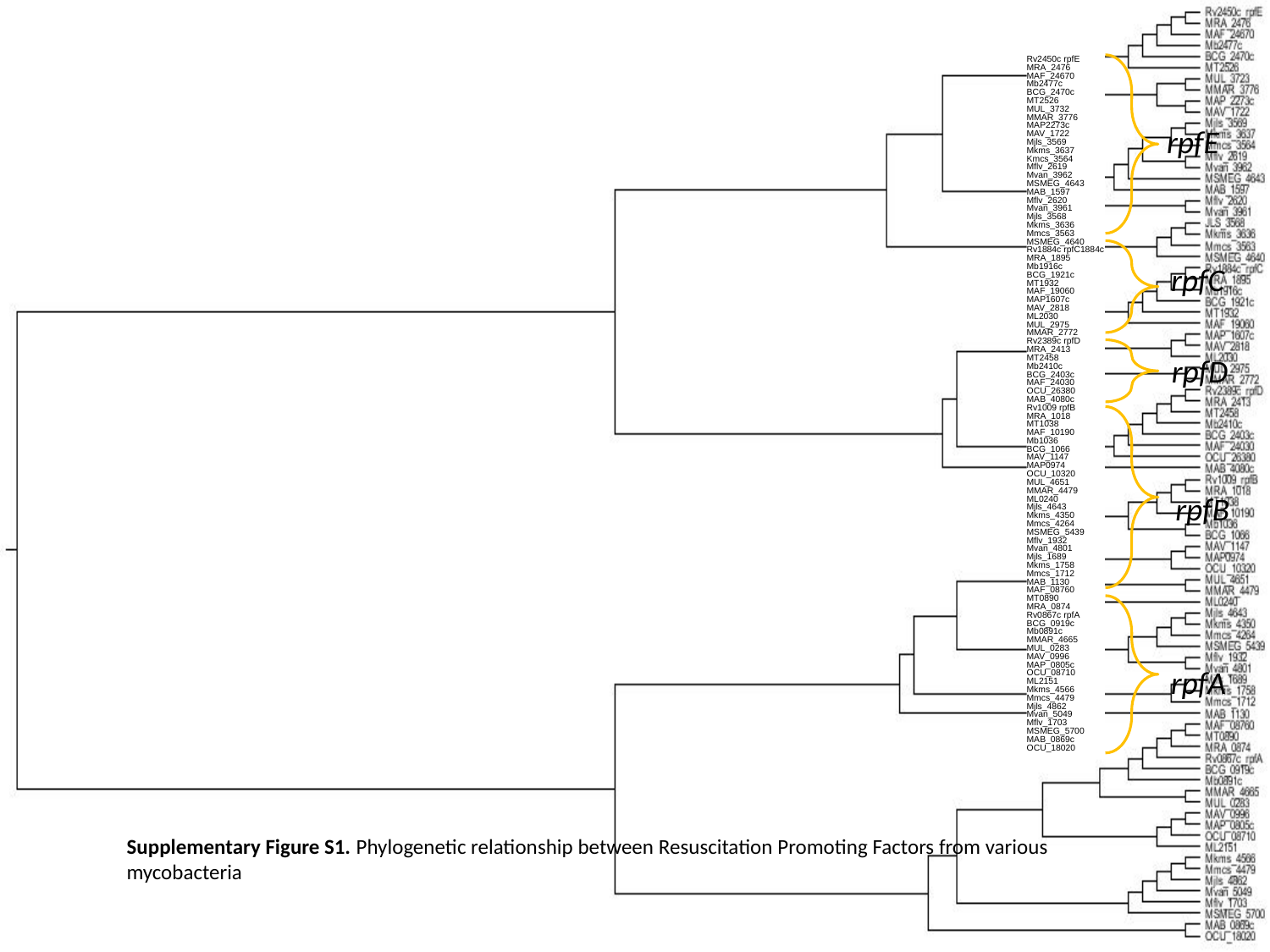

Rv2450c rpfE
MRA_2476
MAF_24670
Mb2477c
BCG_2470c
MT2526
MUL_3732
MMAR_3776
MAP2273c
MAV_1722
Mjls_3569
Mkms_3637
Kmcs_3564
Mflv_2619
Mvan_3962
MSMEG_4643
MAB_1597
Mflv_2620
Mvan_3961
Mjls_3568
Mkms_3636
Mmcs_3563
MSMEG_4640
Rv1884c rpfC1884c
MRA_1895
Mb1916c
BCG_1921c
MT1932
MAF_19060
MAP1607c
MAV_2818
ML2030
MUL_2975
MMAR_2772
Rv2389c rpfD
MRA_2413
MT2458
Mb2410c
BCG_2403c
MAF_24030
OCU_26380
MAB_4080c
Rv1009 rpfB
MRA_1018
MT1038
MAF_10190
Mb1036
BCG_1066
MAV_1147
MAP0974
OCU_10320
MUL_4651
MMAR_4479
ML0240
Mjls_4643
Mkms_4350
Mmcs_4264
MSMEG_5439
Mflv_1932
Mvan_4801
Mjls_1689
Mkms_1758
Mmcs_1712
MAB_1130
MAF_08760
MT0890
MRA_0874
Rv0867c rpfA
BCG_0919c
Mb0891c
MMAR_4665
MUL_0283
MAV_0996
MAP_0805c
OCU_08710
ML2151
Mkms_4566
Mmcs_4479
Mjls_4862
Mvan_5049
Mflv_1703
MSMEG_5700
MAB_0869c
OCU_18020
rpfE
rpfC
rpfD
rpfB
rpfA
Supplementary Figure S1. Phylogenetic relationship between Resuscitation Promoting Factors from various mycobacteria

## Slide 2
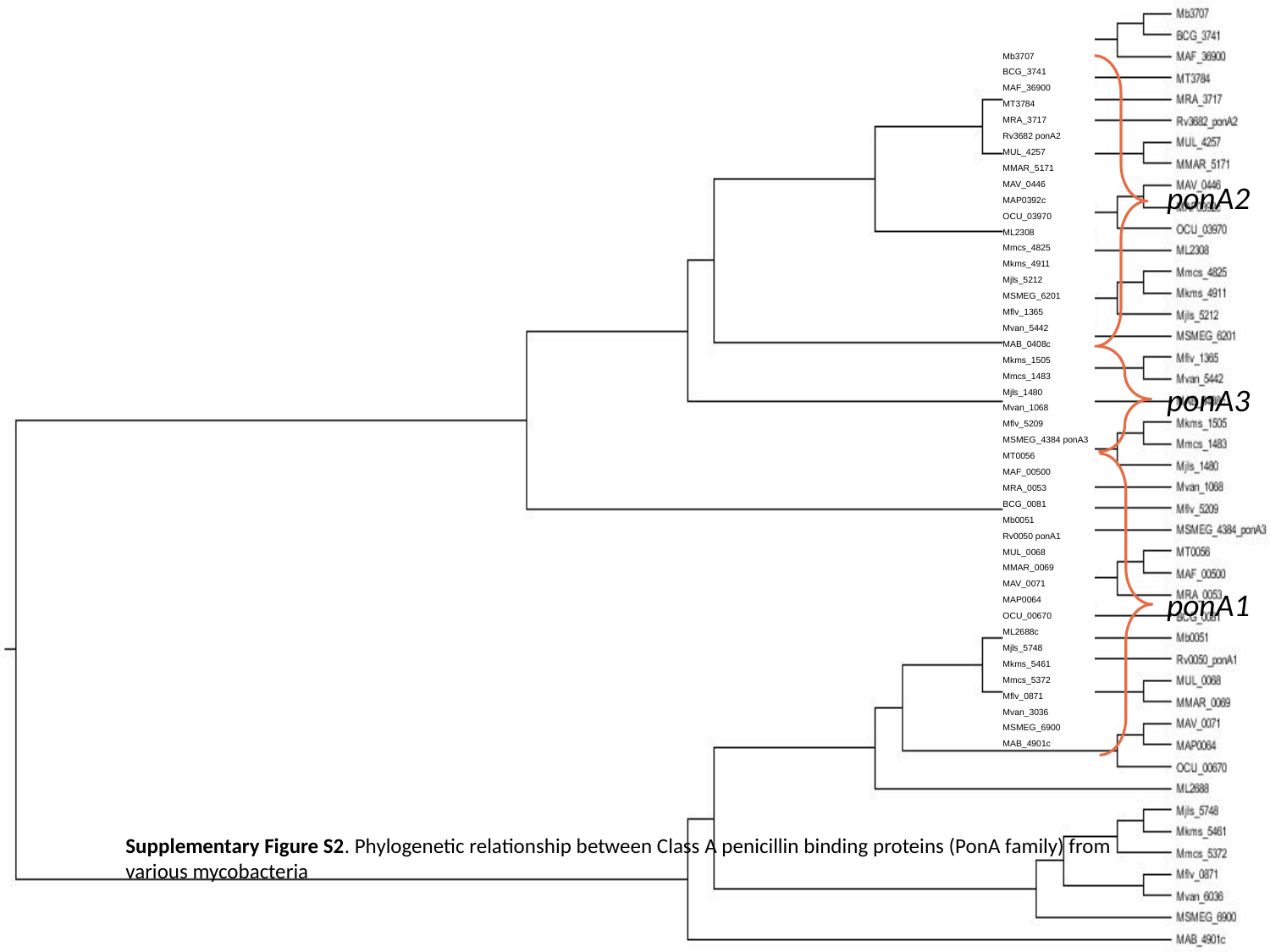

Mb3707
BCG_3741
MAF_36900
MT3784
MRA_3717
Rv3682 ponA2
MUL_4257
MMAR_5171
MAV_0446
MAP0392c
OCU_03970
ML2308
Mmcs_4825
Mkms_4911
Mjls_5212
MSMEG_6201
Mflv_1365
Mvan_5442
MAB_0408c
Mkms_1505
Mmcs_1483
Mjls_1480
Mvan_1068
Mflv_5209
MSMEG_4384 ponA3
MT0056
MAF_00500
MRA_0053
BCG_0081
Mb0051
Rv0050 ponA1
MUL_0068
MMAR_0069
MAV_0071
MAP0064
OCU_00670
ML2688c
Mjls_5748
Mkms_5461
Mmcs_5372
Mflv_0871
Mvan_3036
MSMEG_6900
MAB_4901c
ponA2
ponA3
ponA1
Supplementary Figure S2. Phylogenetic relationship between Class A penicillin binding proteins (PonA family) from various mycobacteria

## Slide 3
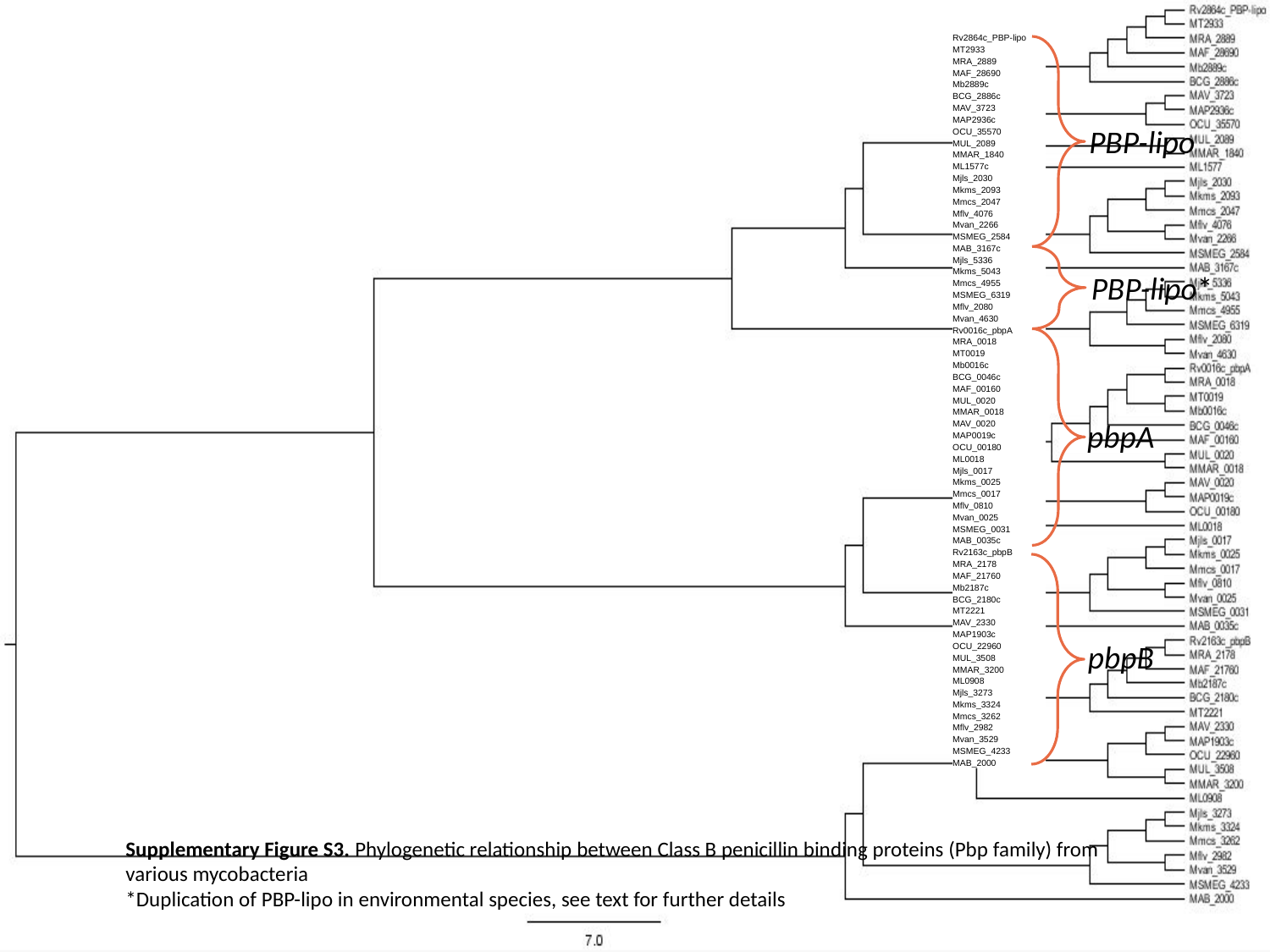

Rv2864c_PBP-lipo
MT2933
MRA_2889
MAF_28690
Mb2889c
BCG_2886c
MAV_3723
MAP2936c
OCU_35570
MUL_2089
MMAR_1840
ML1577c
Mjls_2030
Mkms_2093
Mmcs_2047
Mflv_4076
Mvan_2266
MSMEG_2584
MAB_3167c
Mjls_5336
Mkms_5043
Mmcs_4955
MSMEG_6319
Mflv_2080
Mvan_4630
Rv0016c_pbpA
MRA_0018
MT0019
Mb0016c
BCG_0046c
MAF_00160
MUL_0020
MMAR_0018
MAV_0020
MAP0019c
OCU_00180
ML0018
Mjls_0017
Mkms_0025
Mmcs_0017
Mflv_0810
Mvan_0025
MSMEG_0031
MAB_0035c
Rv2163c_pbpB
MRA_2178
MAF_21760
Mb2187c
BCG_2180c
MT2221
MAV_2330
MAP1903c
OCU_22960
MUL_3508
MMAR_3200
ML0908
Mjls_3273
Mkms_3324
Mmcs_3262
Mflv_2982
Mvan_3529
MSMEG_4233
MAB_2000
PBP-lipo
PBP-lipo*
pbpA
pbpB
Supplementary Figure S3. Phylogenetic relationship between Class B penicillin binding proteins (Pbp family) from various mycobacteria
*Duplication of PBP-lipo in environmental species, see text for further details

## Slide 4
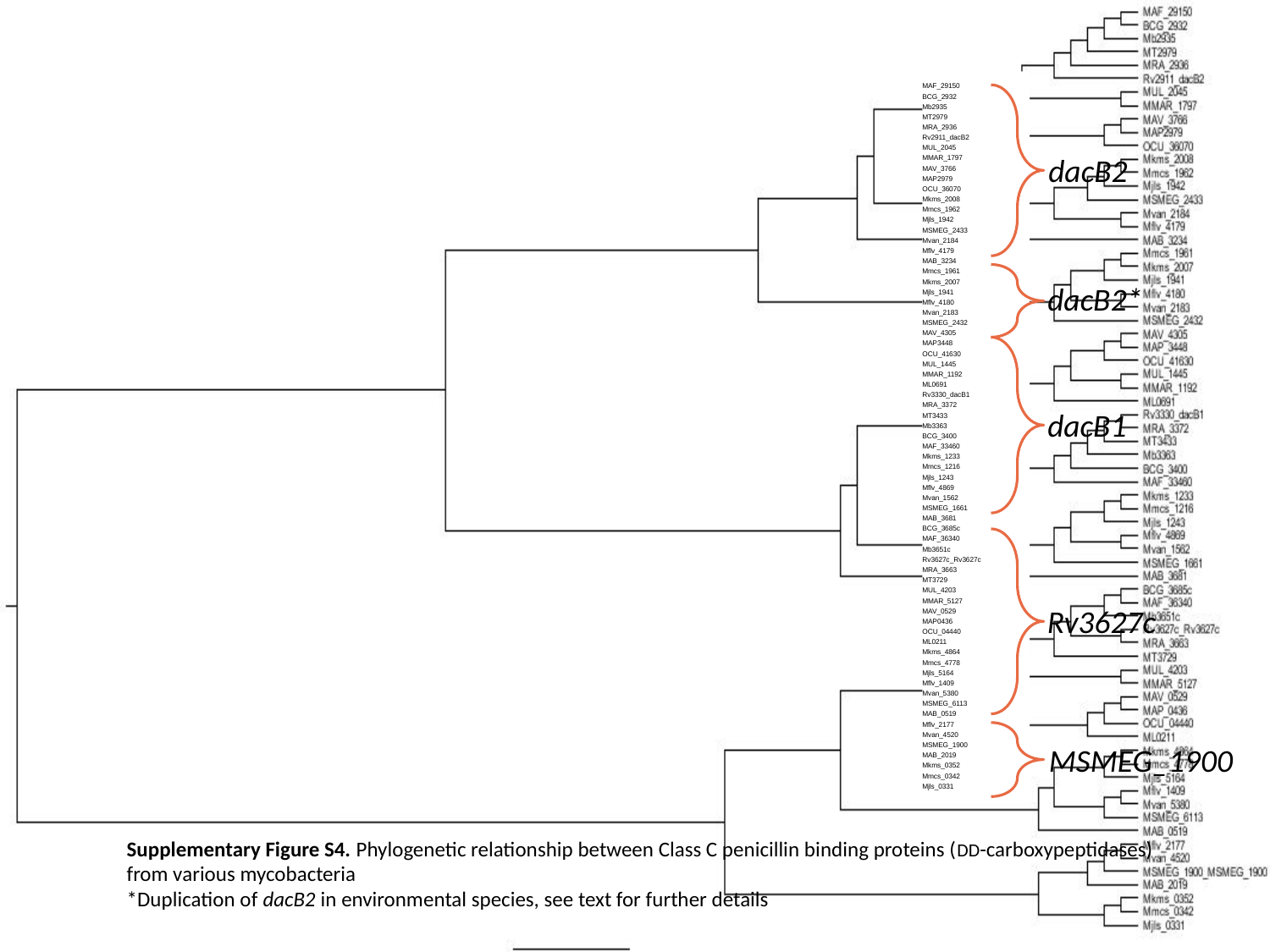

MAF_29150
BCG_2932
Mb2935
MT2979
MRA_2936
Rv2911_dacB2
MUL_2045
MMAR_1797
MAV_3766
MAP2979
OCU_36070
Mkms_2008
Mmcs_1962
Mjls_1942
MSMEG_2433
Mvan_2184
Mflv_4179
MAB_3234
Mmcs_1961
Mkms_2007
Mjls_1941
Mflv_4180
Mvan_2183
MSMEG_2432
MAV_4305
MAP3448
OCU_41630
MUL_1445
MMAR_1192
ML0691
Rv3330_dacB1
MRA_3372
MT3433
Mb3363
BCG_3400
MAF_33460
Mkms_1233
Mmcs_1216
Mjls_1243
Mflv_4869
Mvan_1562
MSMEG_1661
MAB_3681
BCG_3685c
MAF_36340
Mb3651c
Rv3627c_Rv3627c
MRA_3663
MT3729
MUL_4203
MMAR_5127
MAV_0529
MAP0436
OCU_04440
ML0211
Mkms_4864
Mmcs_4778
Mjls_5164
Mflv_1409
Mvan_5380
MSMEG_6113
MAB_0519
Mflv_2177
Mvan_4520
MSMEG_1900
MAB_2019
Mkms_0352
Mmcs_0342
Mjls_0331
dacB2
dacB2*
dacB1
Rv3627c
MSMEG_1900
Supplementary Figure S4. Phylogenetic relationship between Class C penicillin binding proteins (DD-carboxypeptidases) from various mycobacteria
*Duplication of dacB2 in environmental species, see text for further details

## Slide 5
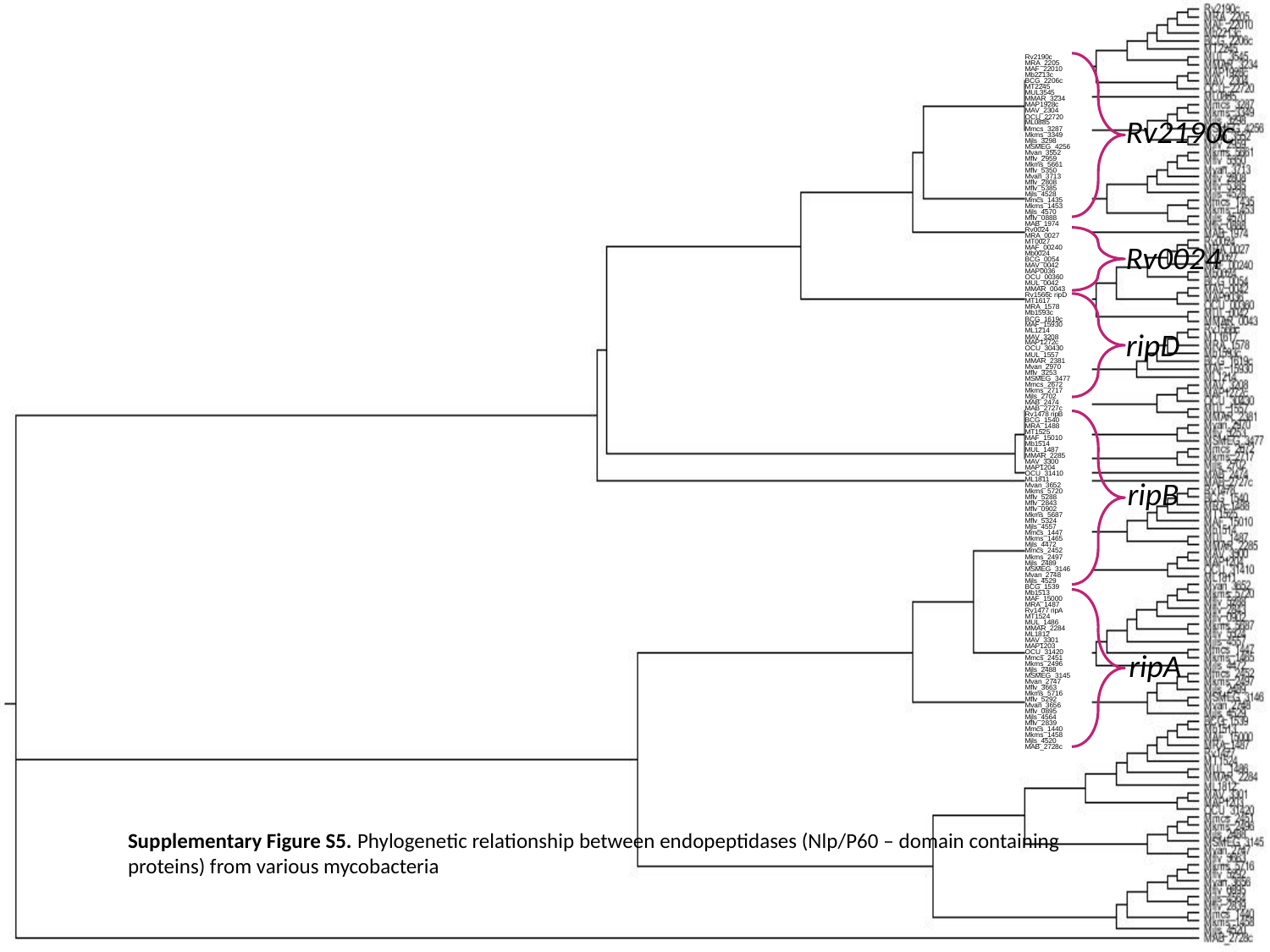

Rv2190c
MRA_2205
MAF_22010
Mb2213c
BCG_2206c
MT2245
MUL3545_
MMAR_3234
MAP1928c
MAV_2304
OCU_22720
ML0885
Mmcs_3287
Mkms_3349
Mjls_3298
MSMEG_4256
Mvan_3552
Mflv_2959
Mkms_5661
Mflv_5350
Mvan_3713
Mflv_2808
Mflv_5385
Mjls_4528
Mmcs_1435
Mkms_1453
Mjls_4570
Mflv_0888
MAB_1974
Rv0024
MRA_0027
MT0027
MAF_00240
Mb0024
BCG_0054
MAV_0042
MAP0036
OCU_00360
MUL_0042
MMAR_0043
Rv1566c ripD
MT1617
MRA_1578
Mb1593c
BCG_1619c
MAF_15930
ML1214
MAV_3208
MAP1272c
OCU_30430
MUL_1557
MMAR_2381
Mvan_2970
Mflv_3253
MSMEG_3477
Mmcs_2672
Mkms_2717
Mjls_2702
MAB_2474
MAB_2727c
Rv1478 ripB
BCG_1540
MRA_1488
MT1525
MAF_15010
Mb1514
MUL_1487
MMAR_2285
MAV_3300
MAP1204
OCU_31410
ML1811
Mvan_3652
Mkms_5720
Mflv_5288
Mflv_2843
Mflv_0902
Mkms_5687
Mflv_5324
Mjls_4557
Mmcs_1447
Mkms_1465
Mjls_4472
Mmcs_2452
Mkms_2497
Mjls_2489
MSMEG_3146
Mvan_2748
Mjls_4529
BCG_1539
Mb1513
MAF_15000
MRA_1487
Rv1477 ripA
MT1524
MUL_1486
MMAR_2284
ML1812
MAV_3301
MAP1203
OCU_31420
Mmcs_2451
Mkms_2496
Mjls_2488
MSMEG_3145
Mvan_2747
Mflv_3663
Mkms_5716
Mflv_5292
Mvan_3656
Mflv_0895
Mjls_4564
Mflv_2839
Mmcs_1440
Mkms_1458
Mjls_4520
MAB_2728c
Rv2190c
Rv0024
ripD
ripB
ripA
Supplementary Figure S5. Phylogenetic relationship between endopeptidases (Nlp/P60 – domain containing proteins) from various mycobacteria

## Slide 6
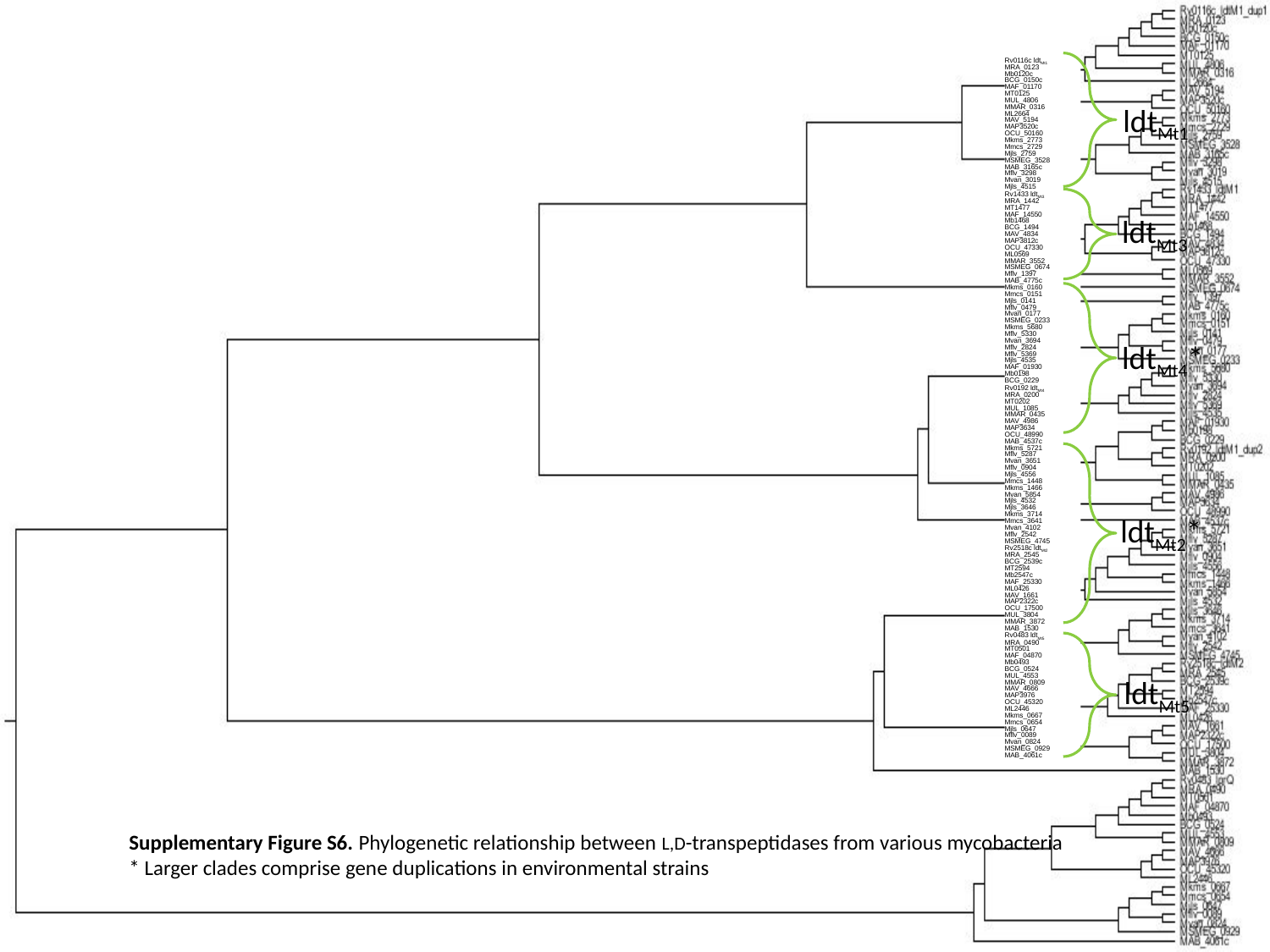

Rv0116c ldtMt1
MRA_0123
Mb0120c
BCG_0150c
MAF_01170
MT0125
MUL_4806
MMAR_0316
ML2664
MAV_5194
MAP3520c
OCU_50160
Mkms_2773
Mmcs_2729
Mjls_2759
MSMEG_3528
MAB_3165c
Mflv_3298
Mvan_3019
Mjls_4515
Rv1433 ldtMt3
MRA_1442
MT1477
MAF_14550
Mb1468
BCG_1494
MAV_4834
MAP3812c
OCU_47330
ML0569
MMAR_3552
MSMEG_0674
Mflv_1397
MAB_4775c
Mkms_0160
Mmcs_0151
Mjls_0141
Mflv_0479
Mvan_0177
MSMEG_0233
Mkms_5680
Mflv_5330
Mvan_3694
Mflv_2824
Mflv_5369
Mjls_4535
MAF_01930
Mb0198
BCG_0229
Rv0192 ldtMt4
MRA_0200
MT0202
MUL_1085
MMAR_0435
MAV_4986
MAP3634
OCU_48990
MAB_4537c
Mkms_5721
Mflv_5287
Mvan_3651
Mflv_0904
Mjls_4556
Mmcs_1448
Mkms_1466
Mvan_5854
Mjls_4532
Mjls_3646
Mkms_3714
Mmcs_3641
Mvan_4102
Mflv_2542
MSMEG_4745
Rv2518c ldtMt2
MRA_2545
BCG_2539c
MT2594
Mb2547c
MAF_25330
ML0426
MAV_1661
MAP2322c
OCU_17500
MUL_3804
MMAR_3872
MAB_1530
Rv0483 ldtMt5
MRA_0490
MT0501
MAF_04870
Mb0493
BCG_0524
MUL_4553
MMAR_0809
MAV_4666
MAP3976
OCU_45320
ML2446
Mkms_0667
Mmcs_0654
Mjls_0647
Mflv_0089
Mvan_0824
MSMEG_0929
MAB_4061c
 ldtMt1
 ldtMt3
 ldtMt4*
 ldtMt2*
 ldtMt5
Supplementary Figure S6. Phylogenetic relationship between L,D-transpeptidases from various mycobacteria
* Larger clades comprise gene duplications in environmental strains

## Slide 7
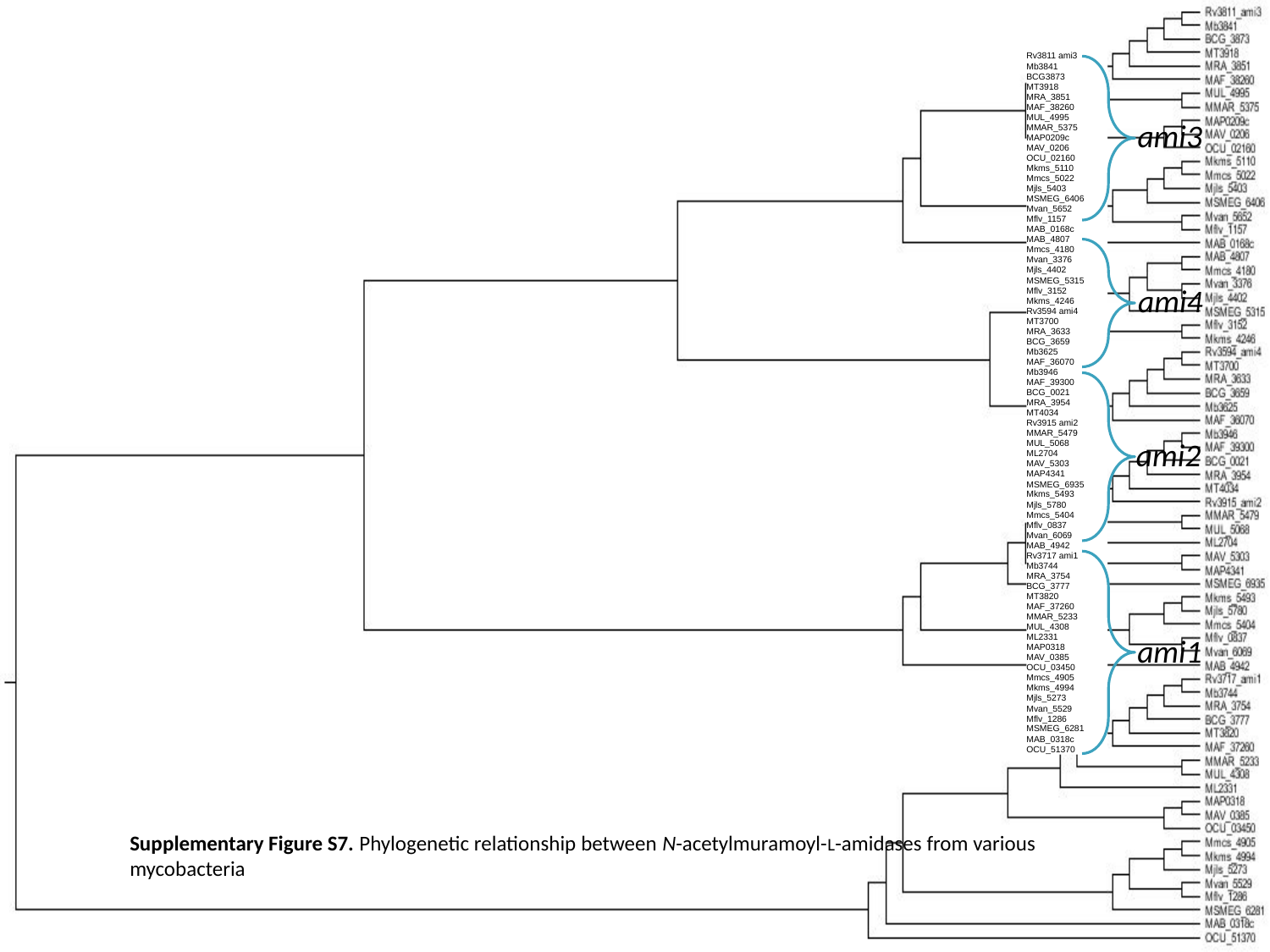

Rv3811 ami3
Mb3841
BCG3873
MT3918
MRA_3851
MAF_38260
MUL_4995
MMAR_5375
MAP0209c
MAV_0206
OCU_02160
Mkms_5110
Mmcs_5022
Mjls_5403
MSMEG_6406
Mvan_5652
Mflv_1157
MAB_0168c
MAB_4807
Mmcs_4180
Mvan_3376
Mjls_4402
MSMEG_5315
Mflv_3152
Mkms_4246
Rv3594 ami4
MT3700
MRA_3633
BCG_3659
Mb3625
MAF_36070
Mb3946
MAF_39300
BCG_0021
MRA_3954
MT4034
Rv3915 ami2
MMAR_5479
MUL_5068
ML2704
MAV_5303
MAP4341
MSMEG_6935
Mkms_5493
Mjls_5780
Mmcs_5404
Mflv_0837
Mvan_6069
MAB_4942
Rv3717 ami1
Mb3744
MRA_3754
BCG_3777
MT3820
MAF_37260
MMAR_5233
MUL_4308
ML2331
MAP0318
MAV_0385
OCU_03450
Mmcs_4905
Mkms_4994
Mjls_5273
Mvan_5529
Mflv_1286
MSMEG_6281
MAB_0318c
OCU_51370
ami3
ami4
ami2
ami1
Supplementary Figure S7. Phylogenetic relationship between N-acetylmuramoyl-L-amidases from various mycobacteria
