# Supplementary material for: Comparative genomics for mycobacterial peptidoglycan remodelling enzymes reveals extensive genetic multiplicity
Source: BMC Microbiol. 2014 Mar 24;14:75. doi: 10.1186/1471-2180-14-75 (PMC3987819; doi:10.1186/1471-2180-14-75)
Supplement: Additional file 2: Table S1 — Mycobacterial strains included in this study. Table S2. Bioinformatics sites used for analysis. [file 1471-2180-14-75-S2.docx]

**Comparative genomics for mycobacterial peptidoglycan remodelling enzymes reveals extensive genetic multiplicity**

**Supplementary Tables**

Edith Erika Machowski, Sibusiso Senzani, Christopher Ealand and Bavesh Kana*

DST/NRF Centre of Excellence for Biomedical TB Research, Faculty of Health Sciences, University of the Witwatersrand, National Health Laboratory Service, Johannesburg, South Africa

[edith.machowski@nhls.ac.za](mailto:edith.machowski@nhls.ac.za), [christopher.ealand@nhls.ac.za](mailto:christopher.ealand@nhls.ac.za), [sibusisu.senzani@nhls.ac.za](mailto:sibusisu.senzani@nhls.ac.za)

*For correspondence: Prof. Bavesh Davandra Kana, DST/NRF Centre of Excellence for Biomedical TB Research, School of Pathology, Faculty of Health Sciences, University of the Witwatersrand and the National Health Laboratory Service, P.O. Box 1038, Johannesburg, 2000, South Africa. Email: [bavesh.kana@nhls.ac.za](mailto:bavesh.kana@nhls.ac.za). Telephone: +27 11 489 9030, Fax: +27 11 489 9397

### Table S1. Mycobacterial strains included in this study.

The NC numbers link directly to the relevant GenBank sites. The Publications link to PubMed at NCBI. Direct Submission indicates that the sequence was deposited at GenBank, and details can be found at the relevant links.

| mycobacterial species / strain | GenBank listing | Publication of genome sequence |
| --- | --- | --- |
| *M. tuberculosis*  H37Rv | [NC_000962](http://www.ncbi.nlm.nih.gov/nuccore/NC_000962) | <http://www.ncbi.nlm.nih.gov/pubmed/20980199> |
| *M. tuberculosis*  H37Ra | [NC_009525](http://www.ncbi.nlm.nih.gov/nuccore/NC_009525) | <http://www.ncbi.nlm.nih.gov/pubmed/18584054> |
| *M. tuberculosis*  CDC1551 | [NC_002755](http://www.ncbi.nlm.nih.gov/nuccore/NC_002755) | <http://www.ncbi.nlm.nih.gov/pubmed/12218036> |
| *M. africanum* GM041182 | [NC_015758](http://www.ncbi.nlm.nih.gov/nuccore/NC_015758) | <http://www.ncbi.nlm.nih.gov/pubmed/22389744> |
| *M. bovis*  AF2122/97 | [NC_002945](http://www.ncbi.nlm.nih.gov/nuccore/NC_002945) | <http://www.ncbi.nlm.nih.gov/pubmed/12788972> |
| *M. bovis* sp. BCG Pasteur 1173P2 | [NC_008769](http://www.ncbi.nlm.nih.gov/nuccore/NC_008769) | <http://www.ncbi.nlm.nih.gov/pubmed/17372194> |
| *M. avium*  104 | [NC_008595](http://www.ncbi.nlm.nih.gov/nuccore/NC_008595) | Direct Submission |
| *M. avium* sub*. paratuberculosis* K-10 | [NC_002944](http://www.ncbi.nlm.nih.gov/nuccore/NC_002944) | <http://www.ncbi.nlm.nih.gov/pubmed/16116077> |
| *M. intracellulare*  ATCC 13950 | [NC_016946](http://www.ncbi.nlm.nih.gov/nuccore/NC_016946) | <http://www.ncbi.nlm.nih.gov/pubmed/22535933> |
| *M. ulcerans*  AGY99 | [NC_008611](http://www.ncbi.nlm.nih.gov/nuccore/NC_008611) | <http://www.ncbi.nlm.nih.gov/pubmed/17210928> |
| *M. marinum*  M | [NC_010612](http://www.ncbi.nlm.nih.gov/nuccore/NC_010612) | <http://www.ncbi.nlm.nih.gov/pubmed/18403782> |
| *M. abscessus*  ATCC19977 | [NC_010397](http://www.ncbi.nlm.nih.gov/nuccore/NC_010397) | <http://www.ncbi.nlm.nih.gov/pubmed/23804391> |
| *M. smegmatis*  mc^2^155 | [NC_008596](http://www.ncbi.nlm.nih.gov/nuccore/NC_008596.) | Direct Submission |
| *M. gilvum*  PYR─GCK | [NC_009338](http://www.ncbi.nlm.nih.gov/nuccore/NC_009338) | Direct Submission |
| *M. vanbaalenii*  PYR─1 | [NC_008726](http://www.ncbi.nlm.nih.gov/nuccore/NC_008726) | Direct Submission |
| *Mycobacterium*  sp. JLS | [NC_009077](http://www.ncbi.nlm.nih.gov/nuccore/NC_009077) | Direct Submission |
| *Mycobacterium*  sp. KMS | [NC_008705](http://www.ncbi.nlm.nih.gov/nuccore/NC_008705) | Direct Submission |
| *Mycobacterium*  sp. MCS | [NC_008146](http://www.ncbi.nlm.nih.gov/nuccore/NC_008146) | Direct Submission |
| *M. leprae*  TN | [NC_002677](http://www.ncbi.nlm.nih.gov/nuccore/NC_002677) | <http://www.ncbi.nlm.nih.gov/pubmed/11234002> |

### Table S2. Bioinformatic sites used for analysis.

For each site is given the link and the PubMed citation at NCBI (http://www.ncbi.nlm.nih.gov/). Where appropriate references for the websites are listed in the main text.

| GenBank | <http://www.ncbi.nlm.nih.gov/genbank/>  <http://www.ncbi.nlm.nih.gov/pubmed/23193287> |
| --- | --- |
| TubercuList | <http://www.ncbi.nlm.nih.gov/pubmed/20980199>  <http://www.ncbi.nlm.nih.gov/pubmed/10376668> |
| GenoList | <http://genodb.pasteur.fr/cgi-bin/WebObjects/GenoList>  <http://www.ncbi.nlm.nih.gov/pubmed/18032431> |
| TBDB | <http://www.tbdb.org>  <http://www.ncbi.nlm.nih.gov/pubmed/18835847> |
| BLAST at NCBI | <http://blast.ncbi.nlm.nih.gov/Blast.cgi>  <http://www.ncbi.nlm.nih.gov/pubmed/2231712> |
| Mycobrowser | <http://mycobrowser.epfl.ch>  <http://www.ncbi.nlm.nih.gov/pubmed/20980200> |
| Artemis Comparison Tool at Sanger | <http://www.sanger.ac.uk/resources/software/act>  <http://www.ncbi.nlm.nih.gov/pubmed/15976072> |
| IMG Webact. | <http://img.jgi.doe.gov/cgi-bin/w/main.cgi?section=Artemis&page=ACTForm>  <http://www.ncbi.nlm.nih.gov/pubmed/22194640> |
| ClustalO | <http://www.ebi.ac.uk/Tools/msa/clustalo>  <http://www.ncbi.nlm.nih.gov/pubmed/21988835>  <http://www.ncbi.nlm.nih.gov/pubmed/20439314> |
| ClustalW2 | <http://www.ebi.ac.uk/Tools/phylogeny/clustalw2_phylogeny> <http://www.ncbi.nlm.nih.gov/pubmed/17846036> |
| FigTree | <http://tree.bio.ed.ac.uk/software/figtree> |
| InterScanPro | <http://www.ebi.ac.uk/Tools/pfa/iprscan>  <http://www.ncbi.nlm.nih.gov/pubmed/11590104> |
| PFAM at Sanger | <http://pfam.sanger.ac.uk>  <http://www.ncbi.nlm.nih.gov/pubmed/22127870> |
| SignalP | <http://www.cbs.dtu.dk/services/SignalP>  <http://www.ncbi.nlm.nih.gov/pubmed/21959131> |
| TMHMM | <http://www.cbs.dtu.dk/services/TMHMM>  <http://www.ncbi.nlm.nih.gov/pubmed/11448883> |
